# Hybrid Graph–Machine Learning Framework for Accurate and Interpretable Band Gap Prediction

**DOI:** 10.1021/acs.jcim.6c00365

**Published:** 2026-03-19

**Authors:** Ayhan Aydın, Ümit Kaya Eryılmaz, Onur Bahattin Alkan, Pınar Kocagöz, Fatih Ekinci, Mehmet Serdar Güzel

**Affiliations:** † Department of Computer Engineering, Faculty of Engineering, 37504 Ankara University, Ankara 06830, Türkiye; ‡ Department of Mathematics, Faculty of Science, Ankara University, Ankara 06100, Türkiye; § Department of Computer Engineering, Faculty of Engineering, Ankara University, Ankara 06830, Türkiye; ∥ Department of Artificial Intelligence and Data Engineering, Faculty of Engineering, Ankara University, Ankara 06830, Türkiye; ⊥ Institute of Artificial Intelligence, Ankara University, Ankara 06100, Türkiye

## Abstract

Accurate prediction of the electronic band gap is essential for accelerating the discovery and design of semiconducting and energy materials. Conventional density functional theory (DFT) methods, while physically rigorous, remain computationally expensive and limited in scalability. In this study, we propose a hybrid artificial intelligence framework that combines graph-based deep learning embeddings with classical machine learning algorithms to achieve high-accuracy, interpretable, and computationally efficient band gap prediction. The model integrates embeddings obtained from CGCNN, MEGNet, and SchNet architectures with physically meaningful crystal descriptors—including maximum electronegativity, crystal system, space group, and spin–orbit coupling—and trains them using optimized gradient-boosting and neural architectures. Trained on 136,000 crystal structures from the Materials Project database, the hybrid model achieves *R* ^2^ = 0.921, MAE = 0.191, and MSE = 0.155, outperforming both classical models (Ward et al., 2016) and standalone graph neural networks such as CGCNN (Xie and Grossman, 2018). The achieved accuracy is statistically comparable to the state-of-the-art ALIGNN model (Choudhary et al., 2021), while requiring lower computational resources and offering enhanced generalization due to the integration of multisource structural information. SHAP-based interpretability analysis highlights that the model captures physically consistent relationships, with metallicity and magnetic site features emerging as dominant factors in band gap prediction. These findings demonstrate that the synergy between deep structural embeddings and classical algorithms provides a powerful, scalable approach for materials informatics. The proposed framework establishes a foundation for multiproperty prediction, transfer learning across databases, and inverse materials design driven by interpretable artificial intelligence.

## Graphical abstract

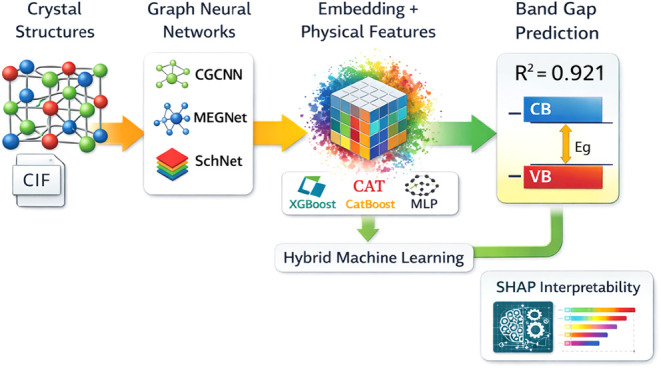

## 1 Introduction

The band gap is one of the most critical parameters that directly determines the electronic, optical, and magnetic properties of materials. Accurate and reliable prediction of the band gap value plays a fundamental role in the discovery and design of next-generation materials, particularly in semiconductors, photovoltaic materials, sensors, and energy storage technologies. Traditionally, band gap predictions have been performed using quantum-mechanics-based approaches such as Density Functional Theory (DFT).^1^ However, such methods pose practical challenges in materials science research due to their high computational cost, limited scalability, and difficulties in processing large databases.

In recent years, artificial intelligence and machine-learning-based methods have emerged as powerful and computationally efficient alternatives for predicting material properties. In particular, graph-based deep learning models (e.g., CGCNN,^2^ MEGNet,^3^ and SchNet^4^) have achieved remarkable performance beyond traditional approaches, owing to their ability to directly learn the topological and chemical relationships of crystals. However, relying solely on graph-based models may limit the generalization capacity and does not always yield optimal results in terms of computational efficiency.

Interpretable machine learning techniques have recently gained significant attention across multiple engineering domains. For example, SHAP-based interpretation has been successfully employed in hybrid deep learning frameworks for predicting material properties such as compressive strength in geopolymer and rubberized concrete systems. These studies demonstrate how explainable AI can improve model transparency and reliability in complex regression tasks. Inspired by this methodological perspective, the present study adopts SHAP analysis to interpret the contribution of structural descriptors and graph-based embeddings in band gap prediction.^5,6^

At this point, integrating embedding vectors derived from graph-based deep learning models with classical machine learning algorithms (such as XGBoost,^7^ Random Forest,^8^ and LightGBM^9^) within a hybrid framework introduces a new paradigm for band gap prediction. This hybrid approach combines the structural representation capability of deep learning with the computational efficiency of classical algorithms, enabling more accurate, interpretable, and scalable predictions.

The rationale for using GNN-derived embeddings as input features for secondary machine learning models is 3-fold. First, it decouples structural representation learning from the final regression stage, which reduces computational cost and avoids end-to-end retraining when additional descriptors or larger data sets are introduced. Second, the two-stage design naturally enables the integration of heterogeneous tabular descriptors (e.g., crystal system, space group, electronegativity, metallicity, and magnetic site information) that are not straightforward to incorporate into a single end-to-end graph architecture. Third, employing tree-based or boosting models in the second stage facilitates transparent feature-attribution analysis (e.g., via SHAP), providing improved interpretability while preserving the rich structural information captured by the GNN embeddings.

The primary objective of this study is to develop a hybrid model that achieves high accuracy and generalizable performance in band gap prediction by synthesizing embedding vectors and prediction scores obtained from graph-based models with classical machine learning approaches. This model is trained on an enriched data set processed through various preprocessing and feature selection techniques, including Mutual Information,^10^ Spearman correlation,^11^ and Principal Component Analysis (PCA).^12^ In this context, the proposed approach aims not only to reduce computational cost but also to accelerate materials discovery processes, thereby creating a broad impact in critical domains, such as electronics, photovoltaics, and energy storage technologies.

## 2 Materials and Methods

In this study, the methodological framework for band gap prediction was designed to encompass data set selection, preprocessing, feature engineering, and modeling stages. Initially, crystal structures obtained from the Materials Project database were used as the foundation. Instead of directly incorporating Density Functional Theory (DFT) outputs into the model, physically meaningful atomic and structural descriptors were preferred. The data set was enriched with additional parameters such as 7 crystal systems, space groups, and Spin–Orbit Coupling (SOC) values, while embedding vectors and prediction scores obtained from graph-based models (CGCNN, MEGNet, SchNet) were provided as inputs to classical machine learning algorithms. Within this framework, the Crystal Graph Convolutional Neural Network (CGCNN) represents crystal structures by treating atoms as nodes and bonds as edges, performing convolution-based feature extraction; the Materials Graph Network (MEGNet) processes atom, bond, and global state information simultaneously through a graph neural network architecture, achieving high accuracy in predicting various physical properties; and the SchNet model integrates interatomic distance information into embedding vectors via continuous-filter convolutions, enabling quantum-mechanical-level prediction accuracy. To enhance model reliability, preprocessing steps such as outlier analysis, missing data cleaning, correlation examination, and mutual-information-based feature selection were applied. In the final stage, the hybrid approach was tested by training several algorithms, including XGBoost, LightGBM, Random Forest, Multi-Layer Perceptron (MLP),^13,14^ Feature Token Transformer (FTT),^15^ and CatBoost,^16^ with hyperparameter optimization performed using the Optuna library.^17^ This comprehensive framework enables a multidimensional evaluation of the band gap prediction performance by assessing the contributions of both graph-based embeddings and physical descriptors.

### 2.1 Data Set and Preprocessing

In this study, crystal structure data were obtained from the Materials Project database via its API. To prevent artificial simplification of the model, columns directly calculated through Density Functional Theory (DFT) were excluded from the data set. Instead, the data set was enriched with atomic and structural descriptors generated using the Magpie framework, along with crystal system and space group information determined through the Pymatgen library.^18^

The data set includes both semiconducting/insulating materials and metallic materials with a band gap value of 0 eV, as obtained from the Materials Project database. These metallic compounds were intentionally retained in the data set in order to preserve the physical diversity of the materials space. No logarithmic transformation was applied to the band gap values since such transformations may introduce numerical instability near zero. Instead, the machine learning models were trained directly on the original band gap values using standard regression loss functions. In addition, the binary feature *is_metal* was included among the input descriptors, allowing the models to distinguish metallic systems from semiconducting and insulating materials during the learning process.

In addition, graph-based models such as CGCNN, MEGNet, and SchNet were executed using the CIF (Crystallographic Information File) of each compound. The intermediate layer (embedding) vectors obtained from these models were subjected to dimensionality reduction using the PCA method, while the band gap values predicted by the graph models were directly incorporated into the machine learning algorithms as numerical inputs. In this way, the structural and electronic information captured by deep-learning-based graph models was intended to enhance the predictive performance of classical machine learning methods.

Additionally, the Spin–Orbit Coupling (SOC) values corresponding to the p-orbitals of the constituent elements were calculated for each crystal, and the mean value was integrated into the model.^18^ The SOC parameters were obtained from the DFTB source,^19^ while estimated values reported in the literature were used for elements not available in the database. The average SOC value was calculated using eq 1

1 $$\mathrm{SO}C_{\mathrm{avg}}=\frac{\left(\sum_{i\in E_{\mathrm{known}}}\left(n_{i}\xi_{i}\right)+\sum_{j\in E_{\mathrm{missing}}}\left(n_{j}{\mathrm{\xi ̂}}_{j}\right)\right)}{\sum_{i}n_{i}}$$

The parameter *n*_*i*_ denotes the number of atoms of the *i*th element in the compound; ξ_*i*_ represents the Spin–Orbit Coupling (SOC) value (in eV) for elements with known tabulated values (*E*_know*n*_); and *ξ̂*_*j*_ corresponds to the estimated SOC value for elements lacking tabulated entries (*E*_missing_).^18^

A comprehensive summary of the fundamental numerical features obtained via the Materials Project API and used in the model training process is presented in Table 1.^20^ This table systematically lists each feature’s definition, physical meaning, and the mathematical constraints that define acceptable value ranges. The *nsites* feature represents the total number of atoms within the unit cell; therefore, negative or noninteger values are considered invalid due to physical constraints. Similarly, parameters such as *volume* (Å^3^) and *density* (g/cm^3^) can only take positive values, with an upper physical limit of approximately 25 g/cm^3^ set for the density. Binary variables such as is_stable, is_gap_direct, and is_metal indicate whether a material is stable, exhibits a direct band gap, or possesses metallic characteristics, and are defined to take only 0 or 1 values. In addition, features such as num_magnetic_sites and num_unique_magnetic_sites specify the number of atoms exhibiting magnetic behavior in the crystal, where negative values have no physical meaning. These constraints play a crucial role in ensuring both the physical consistency and reliability of the data set used for model training. Consequently, Table 1 not only provides a statistical summary but also serves as a foundational reference for the data cleaning and validation process.

**Table 1:** Numerical Features Extracted from the Materials Project Database along with Their Definitions and Physical Constraints^a^

feature name	description	physical limitations
nsites	total number of atoms in the unit cell	0<
volume (Å^3^)	volume of the unit cell	0<
density (g/cm^3^)	density of the material	g/cm^3^<
		25 g/cm^3^>
density_atomic (g/cm^3^)	atomic density	0<
is_stable	thermodynamic stability	0|1
is_gap_direct	whether the electron requires a change in momentum when moving from the valence band to the conduction band	0|1
is_metal	whether the material is metal	0|1
num_magnetic_sites	number of atoms exhibiting magnetic properties in a crystal	0<
num_unique_magnetic_sites	the unique number of atoms in a crystal that exhibit magnetic properties	0<
is_transition_metal	whether the material contains at least one transition metal in its composition	0|1

a Here, the 0|1 constraint indicates that the variable is binary and can take only two possible values, while the < and > constraints denote that the numerical variable assumes values within specific lower and upper bounds.

To enhance the reliability and generalization capability of the model, a comprehensive preprocessing procedure was applied to the data set (Figure 1). In this context, the seven fundamental crystal systems (triclinic, monoclinic, orthorhombic, rhombohedral, tetragonal, hexagonal, and cubic), along with more than 230 different space groups and the ordering_encoded variable, were processed using the target encoding method. The mean band gap value calculated for each category was then incorporated into the data set as a numerical feature.

**Figure 1:**
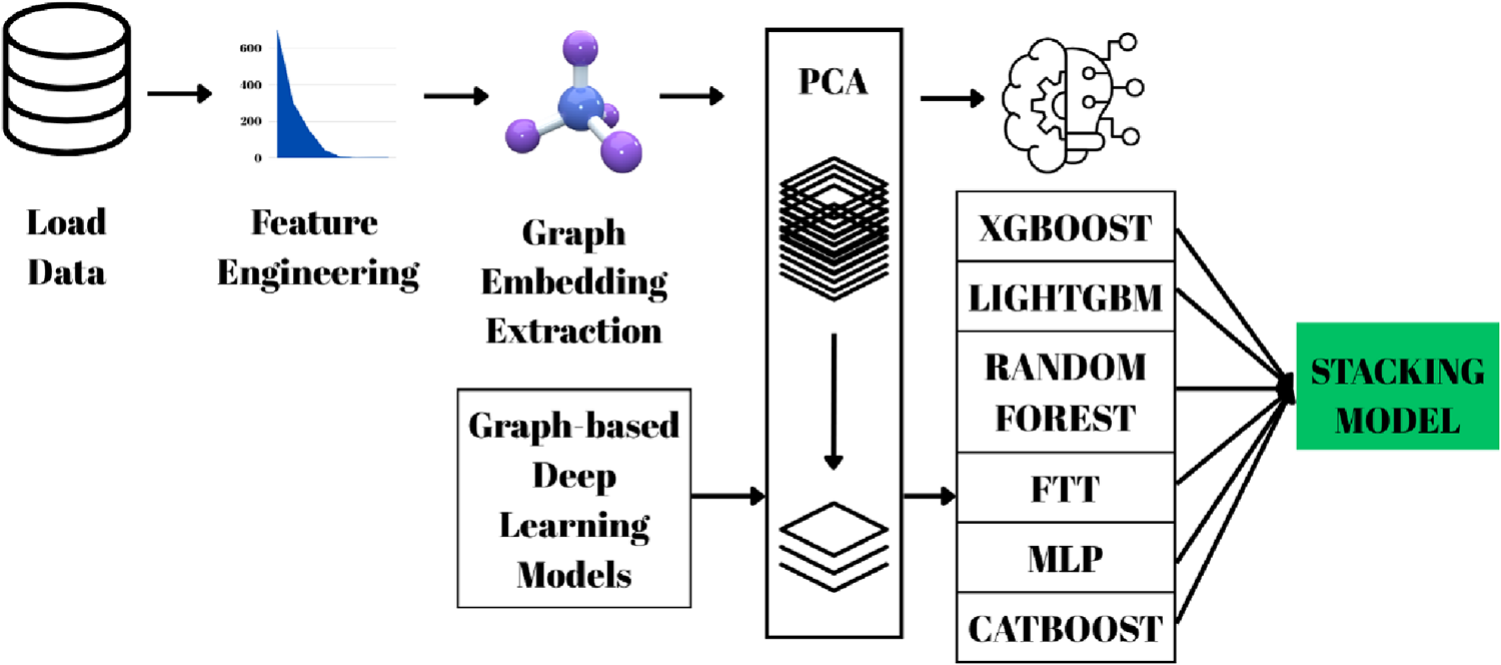
Integrated summary of the data preprocessing and modeling workflow. The process begins with the Data Load step, followed by the Missing Value Analysis stage, where missing values are examined and appropriately handled. Next, the Data Validation step ensures the physical consistency and accuracy of the data.

Outlier analysis was conducted by grouping the features based on the seven crystal systems and statistically examining their minimum, maximum, mean, and standard deviation values. In addition, data distributions were visualized using histogram and Kernel Density Estimation (KDE) plots, and data points that were physically inconsistent or significantly deviated from the overall distribution were removed.^21^ Furthermore, missing (null) values in the data set were handled using appropriate statistical methods, and as a result of these procedures, the final data set was prepared for model training.

In addition to the distribution-based analysis, a practical statistical threshold was applied to the crystal volume feature. Based on the histogram and Kernel Density Estimation (KDE) analysis, the majority of crystal structures were observed within the 0–2500 Å^3^ interval. Structures with volumes outside this range were considered statistical outliers and excluded from the data set in order to prevent extreme values from negatively affecting model stability and generalization performance.

During the feature engineering stage, most physical descriptors were generated using the Magpie feature framework together with data retrieved from the Materials Project database. These frameworks provide descriptor values for a wide range of elements, enabling consistent feature construction for the majority of compounds in the data set.

In cases where certain descriptors were unavailable for rare elements or complex compounds, appropriate statistical preprocessing procedures were applied to ensure data set consistency. Samples with physically inconsistent values were removed during the data validation and outlier analysis steps, while valid compounds with partially missing descriptors were handled by using statistically consistent replacements where necessary. This strategy ensured that rare elements and complex structures were not systematically excluded, while maintaining the reliability of the input features used for model training.

During the Outlier Analysis stage, anomalous values are analyzed, and samples that could negatively impact model performance are corrected or removed. The cleaned data are then numerically transformed using Target Encoding, and in the final stage, Calculate Embeddings and Predictions, embedding vectors are extracted, and prediction operations are performed.

The horizontal axis represents the crystal volume in Å^3^, while the vertical axis indicates its frequency of occurrence within the data set, as shown in Figure 2. In this context, most crystals exhibit volumes in the range of 0–2500 Å^3^. Data points outside this interval were excluded from the data set, as they could negatively affect the model’s performance.

**Figure 2:**
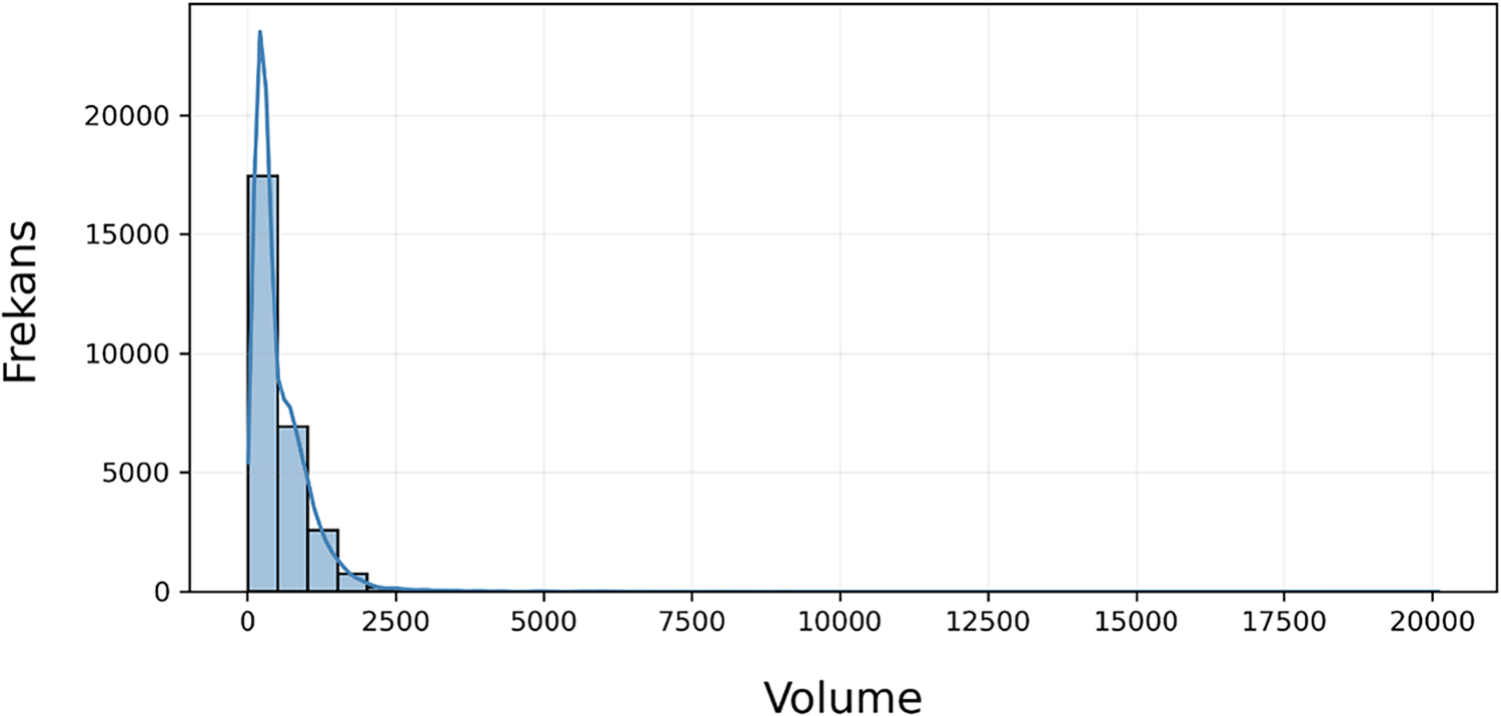
Histogram and Kernel Density Estimation (KDE) plot showing the distribution of the *volume* variable. The histogram represents frequency counts, while the KDE curve illustrates the overall trend of the distribution.

Not all features in the data set carry meaningful information for band gap prediction, either directly or indirectly. Features with insignificant or low information value may lead the model to learn irrelevant patterns (noise), thereby reducing accuracy and increasing computational cost. To prevent this and enhance model interpretability, the entropy-based and model-independent Mutual Information algorithm^22^ was employed (Figure 3). This analysis was particularly applied to the Magpie features, and variables with the highest information values were included in the training set.

**Figure 3:**
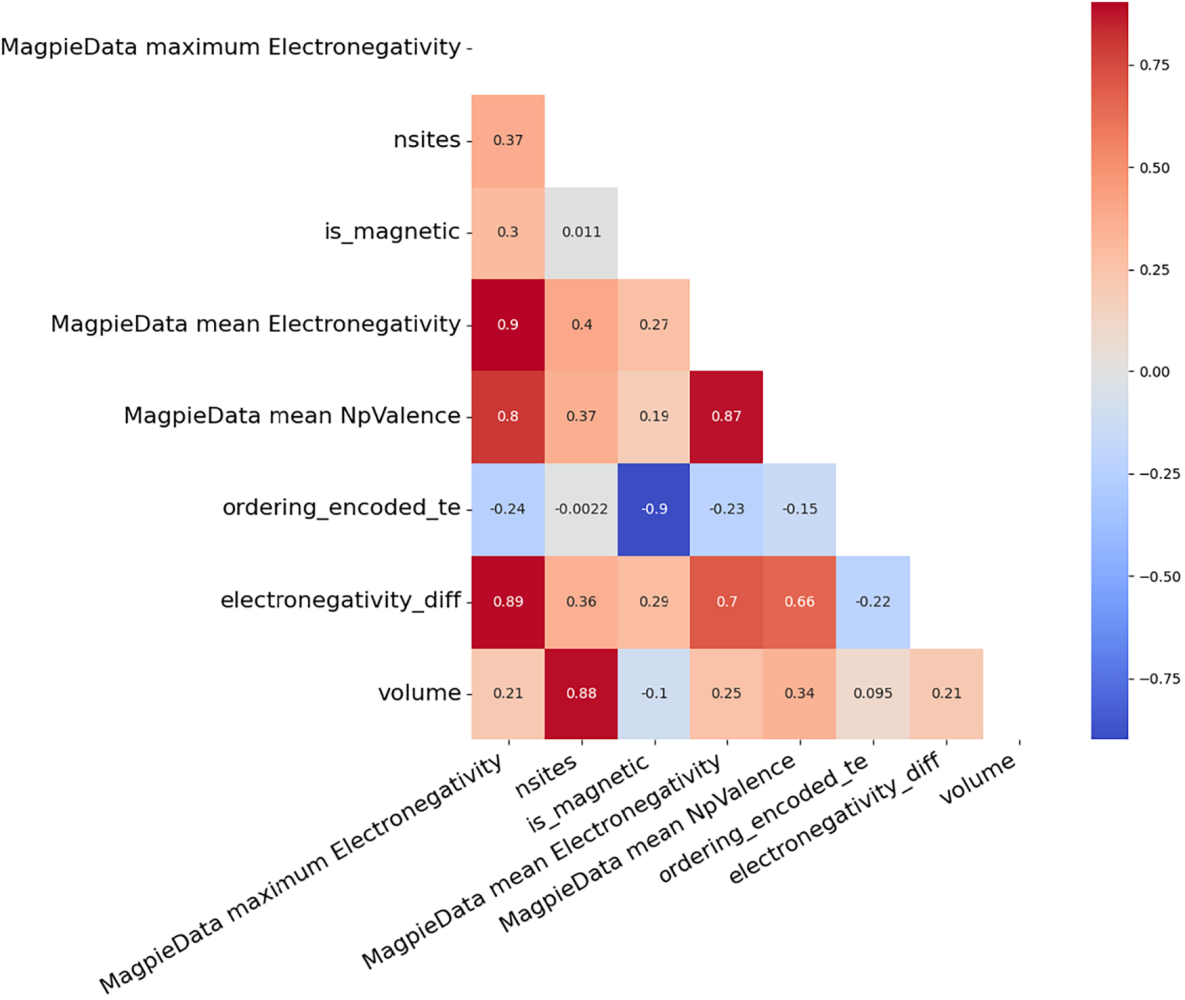
Heatmap of Spearman correlation coefficients illustrating the relationships among the features in the data set. High positive or negative correlation values indicate that the corresponding features exhibit strong nonlinear relationships.

Since highly correlated features can mislead both the mutual information scores and the model learning process, linear and monotonic relationships within the data set were analyzed using Spearman correlation (Figure 3).^23^ Based on this analysis, variables with strong correlations were identified, and features carrying redundant information were either removed from the data set or subjected to dimensionality reduction using the PCA method.^24^

The heatmap illustrating the correlation relationships among the features in the data set is presented in Figure 3. Each cell at the intersection of two variables represents the corresponding correlation coefficient encoded using a color scale. Shades closer to red indicate strong positive correlations between variables, while shades closer to blue represent negative correlations. Based on this analysis, volume, Magpie maximum electronegativity, and ordering_encoded_te were selected for inclusion in the training set due to their high information value and low correlation with other features.^25^

The mutual information scores between the data set features and the target variable, *band* gap, are shown in Figure 4. The mutual information algorithm measures how much knowing one variable reduces the uncertainty of another, effectively capturing not only linear but also nonlinear relationships.^26^ Owing to this property, it operates independently of any specific model and can be reliably used with different learning algorithms. Examination of the results indicates that the strongest relationship with the band gap variable is observed for the is_metal feature, which reflects whether a material is metallic—consistent with the fact that metallic materials typically exhibit near-zero band gaps. To comprehensively evaluate the relationships among variables, the Mutual Information method was used alongside the Spearman correlation, which is commonly applied to measure linear and monotonic associations.^27^ Unlike traditional correlation measures, Mutual Information provides a more holistic perspective of the data set by capturing both linear and nonlinear dependencies, thereby revealing complex relationships that classical methods may overlook.^28^ Additionally, a Random-Forest-based feature importance analysis was incorporated to better expose nonlinear effects and uncover multidimensional information that the model could potentially learn. Based on these analyses, the following variables from the MagpieData data set were selected for inclusion in the model: maximum electronegativity, nelements, volume, density, density_atomic, is stable, is_gap_direct, is_metal, is_magnetic, num_unique_magnetic_sites, spacegroup_symbol, crystal_system, and ordering.

**Figure 4:**
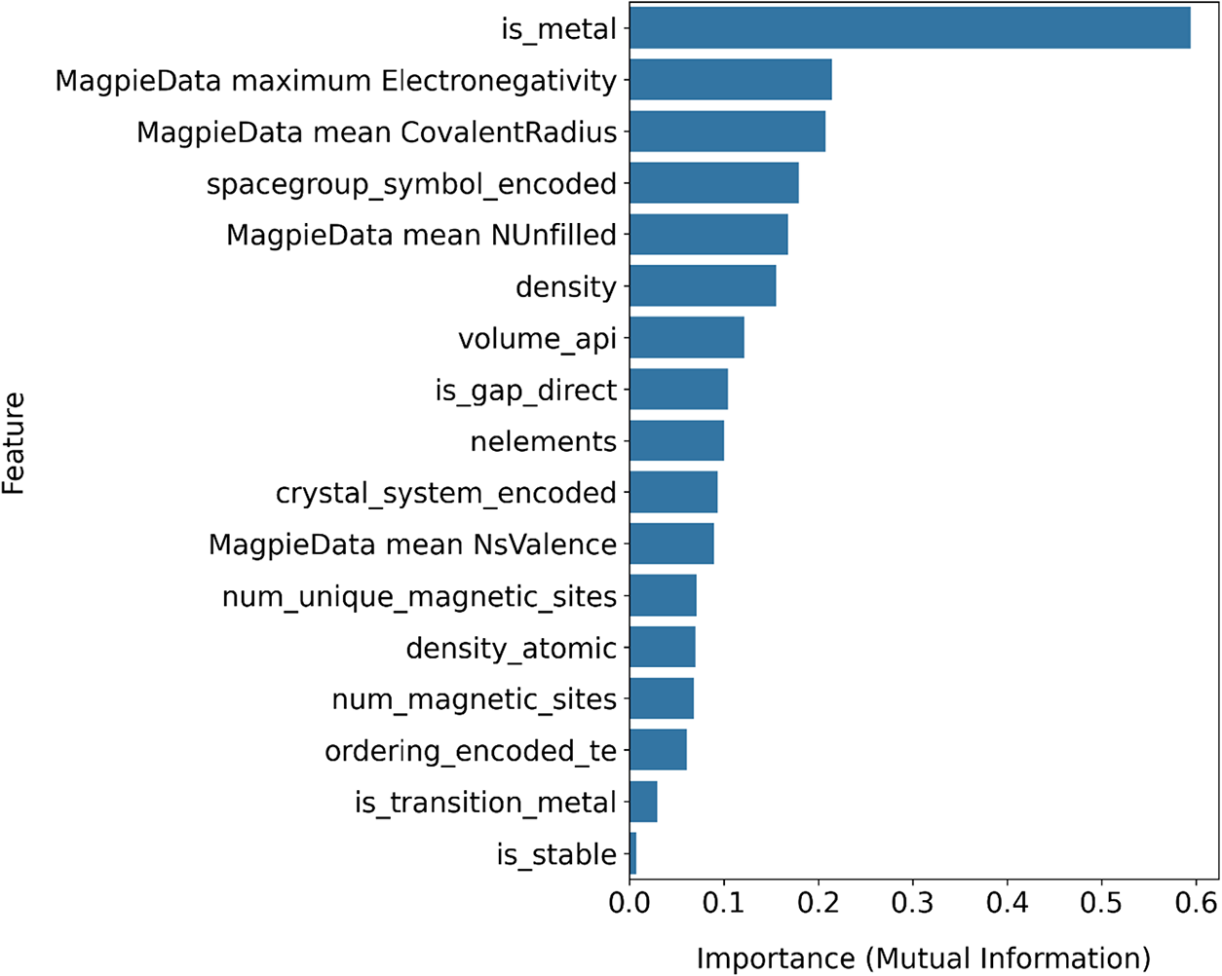
Mutual information scores illustrating the relationship between the data set features and the target variable, *band gap*. Higher scores indicate that the corresponding features play a more significant role in band gap prediction, while lower scores suggest comparatively weaker contributions.

### 2.2 Model Training

In this study, the Optuna library was employed for hyperparameter optimization due to its flexible structure and pruning feature, which reduces unnecessary trials.^29^ This approach aimed to efficiently achieve optimal results across wide and complex parameter spaces.

The SchNet and CGCNN models were trained using CIF files downloaded from the Materials Project database.^20^ In SchNet, the embedding vectors were extracted from the “self.embedding” layer, where atomic identities are meaningfully represented. In CGCNN, the embedding vectors were obtained from the fully connected layer preceding the final prediction layer.^30^ The MEGNet model was utilized in its pretrained form, and embedding extraction was performed from the “concatenate_1” layer of the official GitHub repository’s *Bandgap_MP_2018* model, as this layer integrates multiple information streams (Figure 5).^3^ From each graph model, both the embedding vectors and the predicted band gap values were obtained and incorporated into the training data set.^20,3^

**Figure 5:**
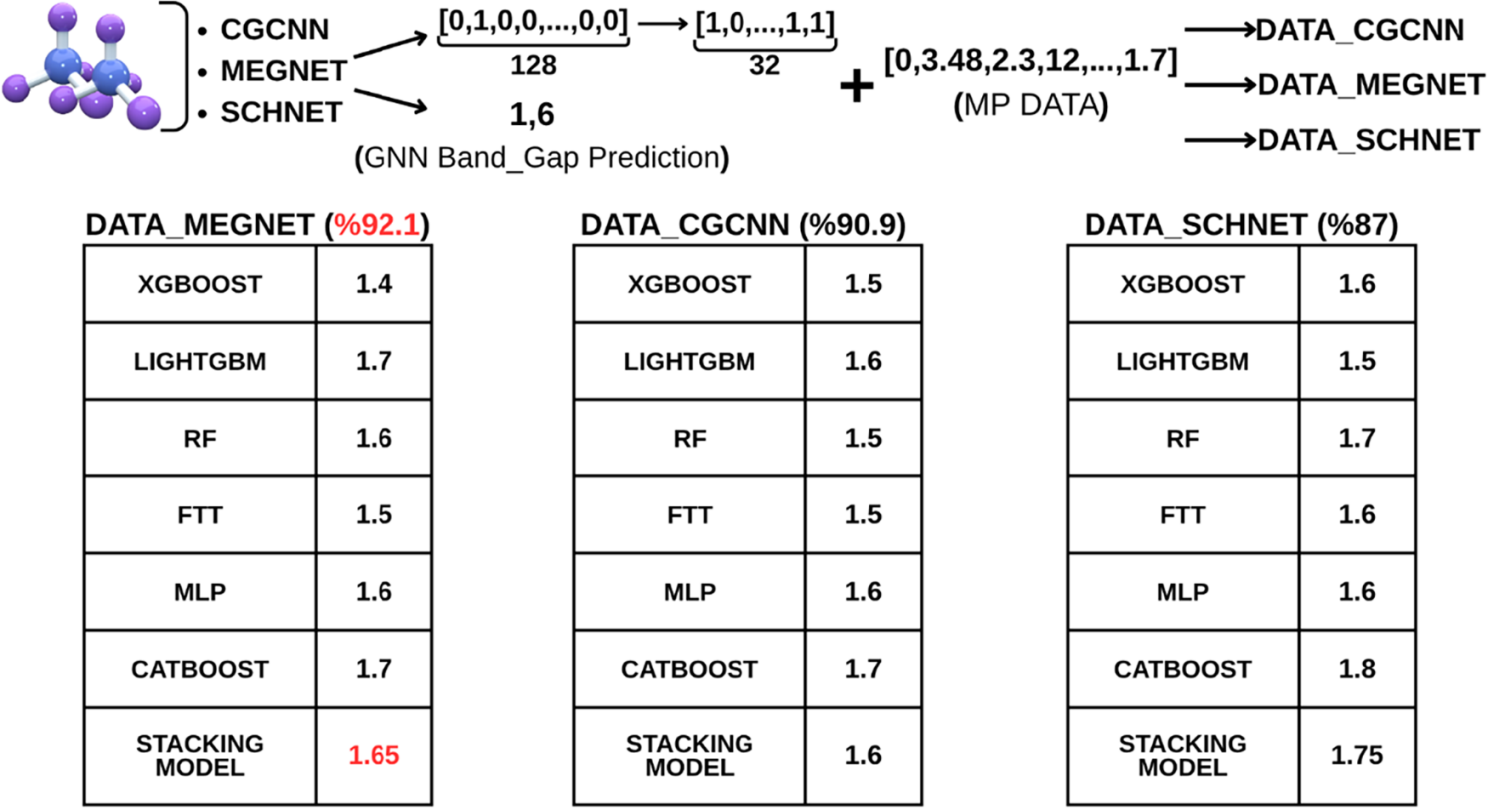
Flow diagram illustrating the model training process combining GNN-based embeddings and band gap predictions with Materials Project data, followed by training six machine learning algorithms and a final stacking ensemble model.

The final feature space consisted of a combination of physically meaningful crystal descriptors and graph-based representations derived from the CGCNN, MEGNet, and SchNet models. Specifically, the input features included selected Magpie descriptors such as maximum electronegativity, number of elements, crystal volume, density, atomic density, stability indicator (is_stable), direct band gap indicator (is_gap_direct), metallicity (is_metal), magnetic information (is_magnetic, num_unique_magnetic_sites), crystal system, space group, and ordering parameters. In addition to these physical descriptors, the band gap predictions produced by the graph neural network models were also included as numerical input features. Since the embedding vectors generated by the graph models are typically high-dimensional, Principal Component Analysis (PCA) was applied to reduce dimensionality before incorporating them into the machine learning models.

The obtained embedding vectors (dimensionally reduced using PCA) and predicted values were incorporated as inputs into machine learning and deep learning algorithms such as XGBoost, LightGBM, Random Forest, FTT, MLP, and CatBoost.^31–36^ The hyperparameters of the tree-based models were optimized using the Optuna algorithm.^37^

For the XGBoost model, the Optuna optimization process explored several key hyperparameters, including the number of estimators, learning rate, maximum tree depth, subsample ratio, and column sampling rate. The search ranges were defined broadly to allow sufficient exploration of the parameter space, with the learning rate varying between **0.01** and **0.3**, the maximum tree depth between **3** and **12**, and the number of estimators between **100** and **1000**.

For the Random Forest model, the optimization included parameters such as the number of trees, maximum tree depth, minimum sample per split, and minimum sample per leaf. The explored ranges included **100–1000 trees**, maximum depths between **5** and **30**, and varying minimum sample constraints to balance model complexity and generalization performance.

Model performance was evaluated using **10-fold cross-validation**, where the data set was randomly partitioned into ten subsets and each subset was used once as the validation set. Because the data set contains approximately **136,000 crystal structures** spanning a wide range of chemical compositions, crystal systems, and space groups, each fold includes a representative distribution of structural families. Furthermore, the model relies on graph-based embeddings and physically meaningful descriptors rather than direct structural identifiers, which reduces the risk of overoptimal results caused by structurally similar materials appearing in both training and validation subsets.

For each model, performance metrics including *R* ^2^ (Coefficient of Determination), MAE (Mean Absolute Error), and MSE (Mean Squared Error) were calculated, accompanied by error distribution plots and predicted–actual value comparison graphs.^32^ The performance tables of the best-performing models trained on data sets derived from the three different graph models are presented in the Results Section.

## 3 Results

Six models trained using the embeddings and prediction scores extracted from the MEGNet model, along with their corresponding results, are presented in Table 2. As observed, the best-performing individual model was XGBoost, achieving an *R* ^2^ score of 92.1%. Additionally, several stacking (ensemble) models were trained by combining different algorithms, and the most successful stacking model—formed by integrating the FTT, MLP, and CatBoost models under a linear regression framework—achieved an *R* ^2^ of 92.1%, MAE of 0.191, and MSE of 0.15.

**Table 2:** Models Trained on the Data Set Generated Using the MEGNet Model and Their Corresponding Results

MEGNET	*R* ^2^	MAE	MSE
XGBOOST	0.921	0.197	0.159
LIGHTGBM	0.919	0.193	0.161
RANDOM FOREST	0.889	0.269	0.220
FTT	0.914	0.192	0.171
MLP	0.910	0.208	0.179
CATBOOST	0.919	0.198	0.161
STACKING MODEL	0.921	0.191	0.155

Although certain chemical elements or structural families may appear less frequently in the data set, the proposed framework is designed to mitigate potential bias arising from such sparsity. The model does not rely solely on elemental identity but instead learns from graph-based structural representations together with physically meaningful descriptors such as electronegativity, density, crystal system, space group, and magnetic characteristics. These representations encode general atomic environments and bonding patterns, enabling the model to capture transferable structure–property relationships. Consequently, materials with similar structural environments can still be effectively represented in the feature space even when specific elements or compositions are sparsely represented in the training data set.

When the prediction performance of the model is examined based on error distribution (Figure 6), the horizontal axis represents the model’s predicted values, while the vertical axis shows the difference between the predicted and actual values. The red line corresponds to the Error = 0 reference, indicating perfect predictions. In an ideal scenario, data points are expected to cluster around this line, and a similar pattern is observed in the analyzed model. The analysis revealed that 75% of the predictions fall below an error value of 0.240 eV, and 90% fall below 0.531 eV, providing valuable insights into the model’s error characteristics.

**Figure 6:**
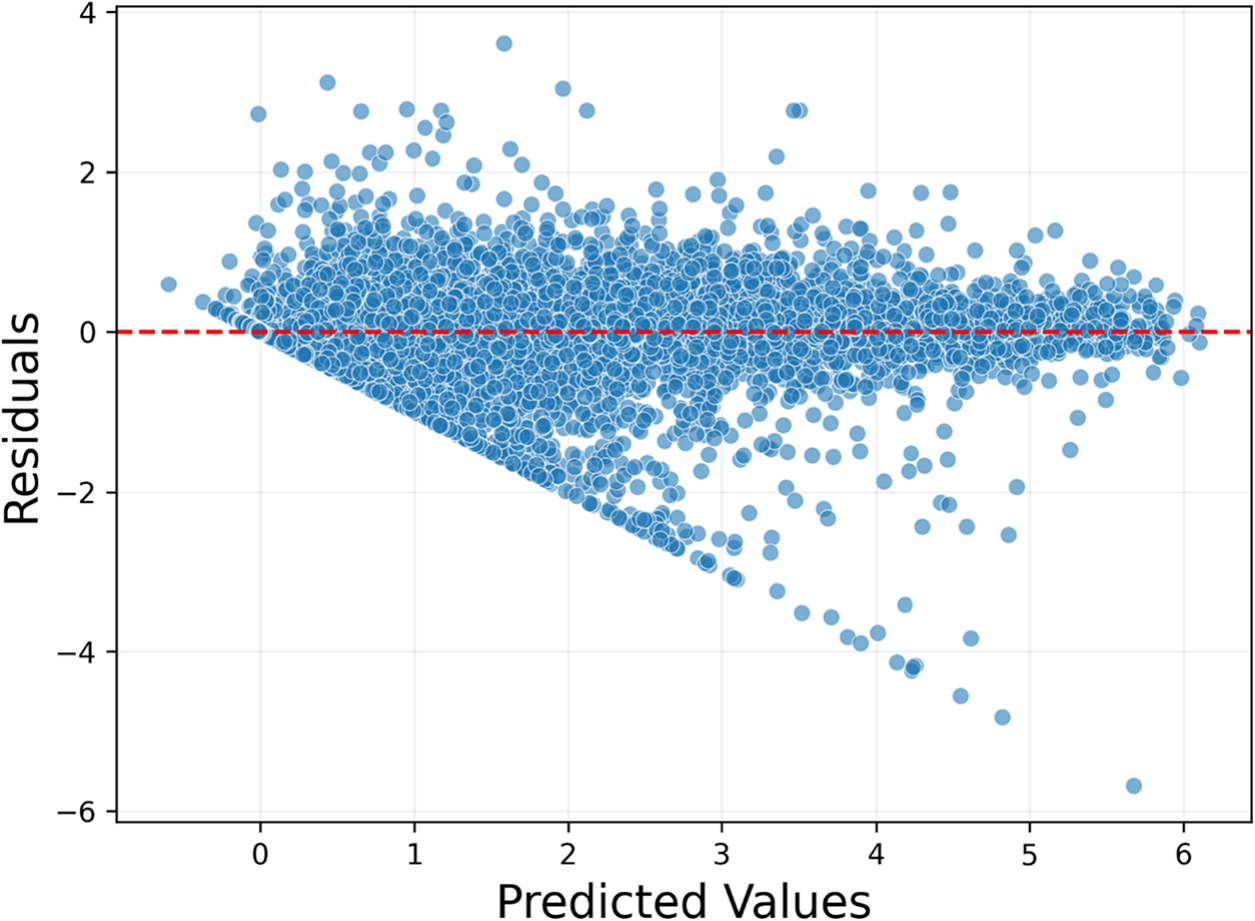
Error distribution of the stacking model. Most data points are clustered around the red line, indicating that the model’s predictions are generally accurate and the error levels remain low.

To evaluate the model’s prediction performance, the horizontal axis represents the predicted values of the composite model, while the vertical axis shows the actual values (Figure 7). The red diagonal line, corresponding to *Prediction = Actual*, represents the ideal scenario in which the model’s predictions perfectly match the true values. In this context, a well-performing model is expected to exhibit a dense clustering of data points along this diagonal. The alignment between predicted and actual values indicates a consistent relationship between the model’s outputs and the true band gap values. However, it was observed that around the region where the actual band gap equals zero, the model produced positive errors of up to 5.2 eV. Calculations revealed that the mean of positive errors was 0.20 eV, while the mean of negative errors was 0.34 eV. When the weighted average of the total prediction count was considered, the resulting 0.002 eV indicated that the model does not exhibit any significant bias toward positive or negative error tendencies.

**Figure 7:**
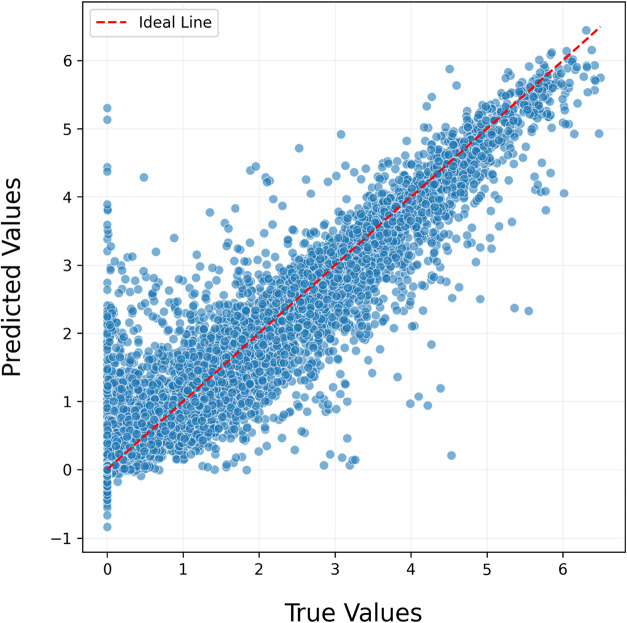
Predicted versus actual value plot of the stacking model, illustrating the alignment between the model’s predictions and the true values. Most data points are concentrated around the reference line, indicating that the model demonstrates a strong predictive capability and produces results close to the actual values.

Understanding which variables the model learns from is a crucial step in interpreting how the model functions and identifying which features have a greater influence on the band gap. However, since the stacking model learns from the outputs of other models, such analyses cannot directly reveal the individual feature contributions within it. Therefore, the interpretability analysis was performed by using the XGBoost model, which was the best-performing individual model outside the ensemble. The SHAP (SHapley Additive exPlanations) summary plot for the XGBoost model is presented in Figure 8. In the SHAP plot, the feature at the top has the greatest influence on the model. A concentration of red points toward positive SHAP values indicates an increase in the prediction, while a concentration of blue points in the same region suggests a decrease. Conversely, when red and blue points are positioned on opposite sides of the SHAP value axis, this implies a positive or negative correlation, respectively.

**Figure 8:**
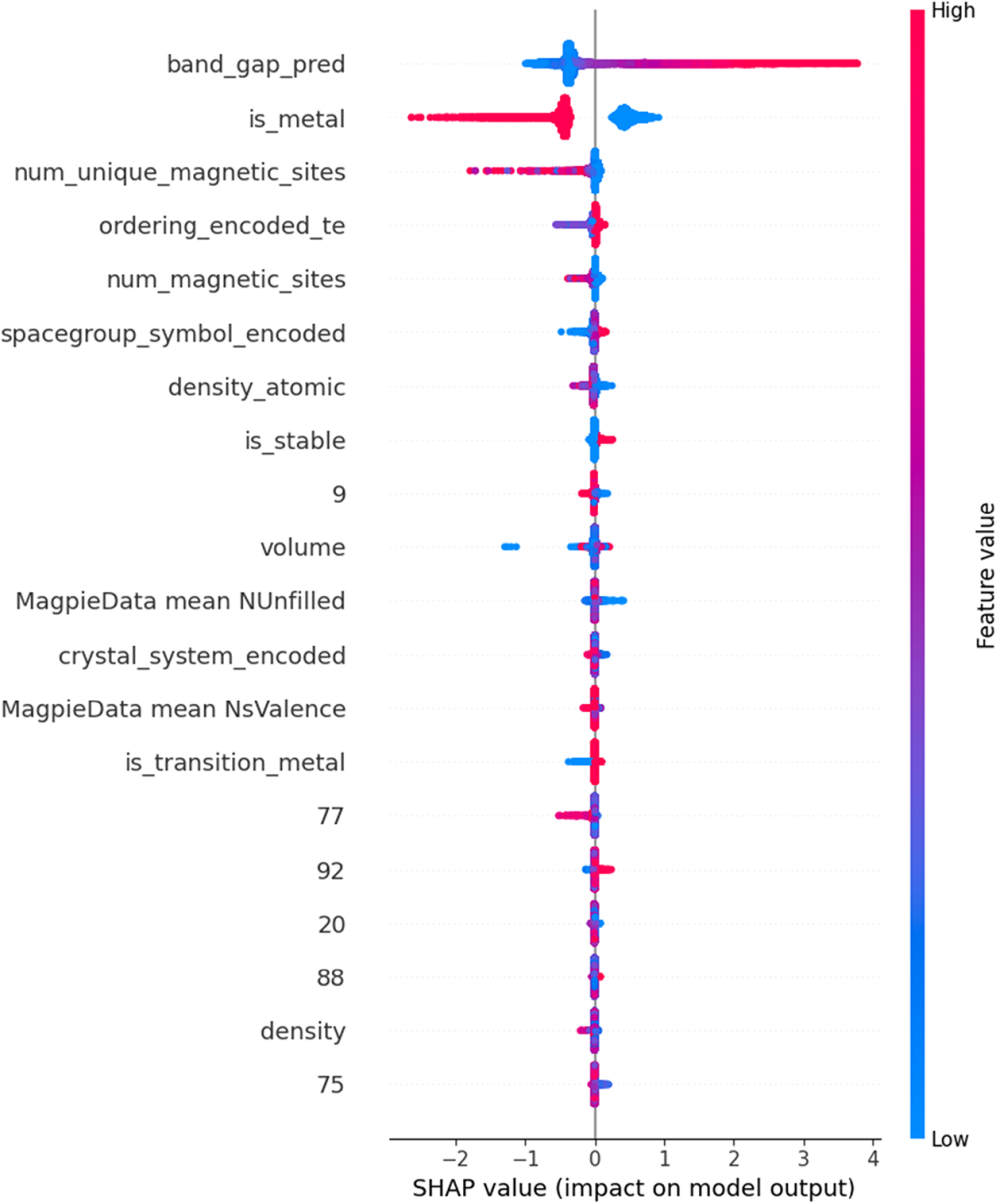
SHAP summary plot illustrating the relative importance of the input features in the band gap prediction model. The horizontal axis represents the **SHAP value**, which quantifies the contribution of each feature to the predicted band gap value. Positive SHAP values indicate that the feature increases the predicted band gap, while negative values indicate a decreasing effect. The vertical axis lists the input features ranked according to their overall importance in the model. Each point represents an individual sample from the data set, and the color scale reflects the magnitude of the feature value (red indicating higher values and blue indicating lower values).

The features that the model assigns the greatest importance are shown in Figure 8. The most critical factors were identified as the band gap prediction generated by the MEGNet model and the information indicating whether the material is metallic. The analysis revealed a positive correlation between the band gap values predicted by the MEGNet model and those predicted by the developed model, whereas a negative correlation was observed between the model’s predictions and the material’s metallic nature. The numerically labeled features in the plot correspond to the embedding outputs obtained from the MEGNet model.

The six models trained using the embeddings and prediction scores extracted from the CGCNN model, along with their corresponding results, are presented in Table 3. As observed, the best-performing model was FTT (Feature Token Transformer), achieving an *R* ^2^ score of 90.6%.

**Table 3:** Models Trained on the Data Set Generated Using the CGCNN Model and Their Corresponding Results

CGCNN	*R* ^2^	MAE	MSE
XGBOOST	0.906	0.219	0.195
LIGHTGBM	0.901	0.223	0.190
RANDOM FOREST	0.896	0.238	0.200
FTT	0.893	0.228	0.206
MLP	0.898	0.229	0.170
CATBOOST	0.905	0.226	0.183
STACKING MODEL	0.909	0.213	0.175

When the error distribution of the model is examined (Figure 9), the horizontal axis represents the predicted values, while the vertical axis shows the difference between the predicted and actual values. The red dashed line in the graph indicates zero error, and the clustering of data points around this line reflects the model’s consistency. The analysis revealed that 75% of the predictions had errors below 0.288 eV, and 90% had errors below 0.614 eV, providing important insights into the model’s error behavior. Additionally, predictions within the 0–4.5 eV range exhibited a wider spread, with 75% of errors below 0.383 eV and a mean absolute error (MAE) of 0.273 eV. In contrast, within the 4.5–6 eV range, 75% of errors were below 0.451 eV, and the MAE was calculated as 0.389 eV, indicating that the model performs better in the 0–4.5 eV range.

**Figure 9:**
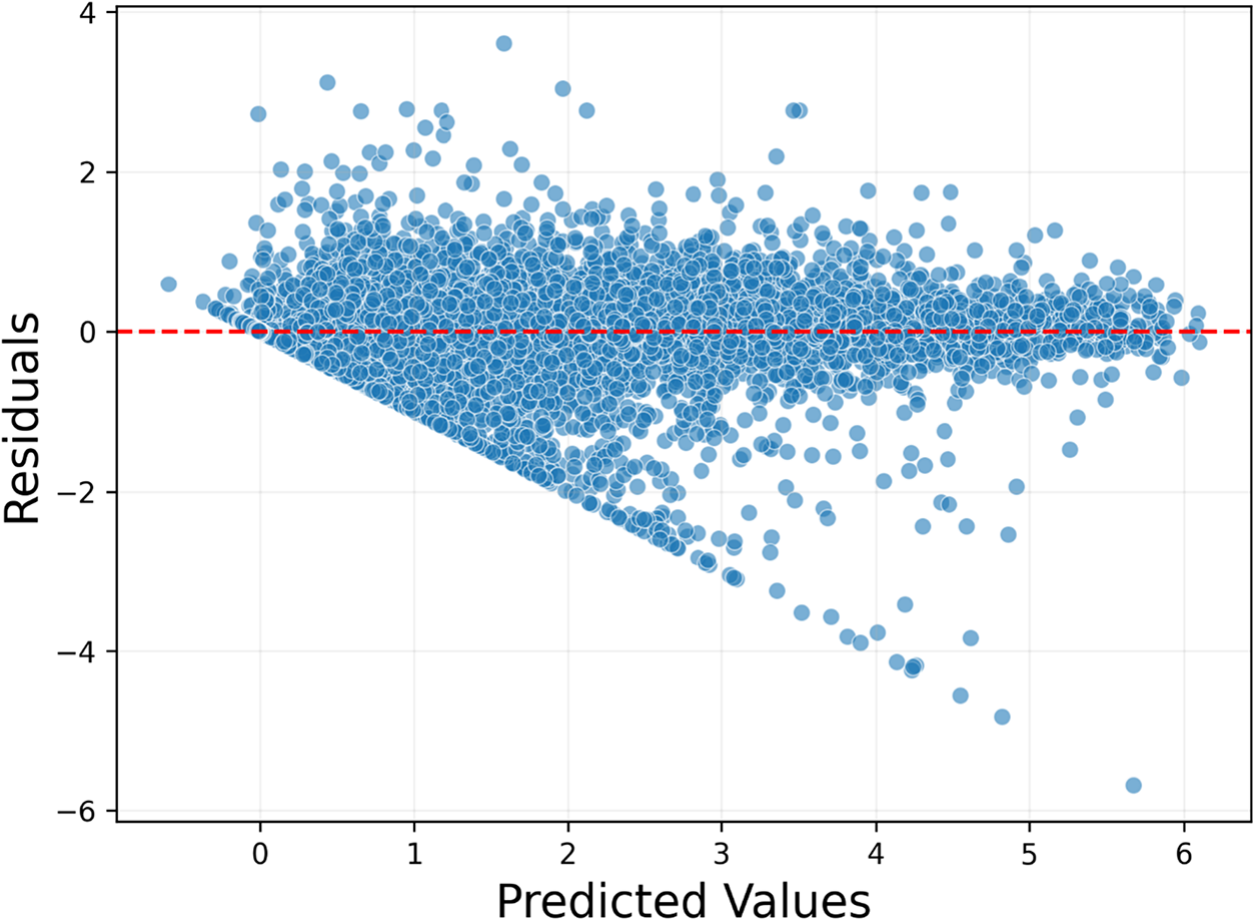
Residuals vs predicted plot of the stacking model, illustrating the distribution of predicted values against residuals. The concentration of data points around the red reference line indicates that the model generally produces balanced predictions and that the errors are, on average, close to zero.

The model’s predictive performance was evaluated based on the relationship between the predicted and actual values (Figure 10). In the graph, the horizontal axis represents the model’s predicted values, while the vertical axis shows the actual values. The red dashed diagonal line corresponds to the *Prediction = Actual* line, representing the ideal scenario in which the model’s predictions perfectly match the true values. In this context, a well-performing model is expected to exhibit a dense clustering of data points along this diagonal. The alignment between predicted and actual values indicates a consistent relationship between the model’s outputs and the true band gap values. However, it was observed that around the region where the actual band gap equals zero, the model produced positive errors of up to 5.2 eV. The calculations revealed that the mean of positive errors was 0.206 eV, while the mean of negative errors was 0.391 eV. When the weighted average of the total prediction count was considered, the resulting value of 0.0026 eV indicated that the model does not exhibit any significant bias toward positive or negative errors.

**Figure 10:**
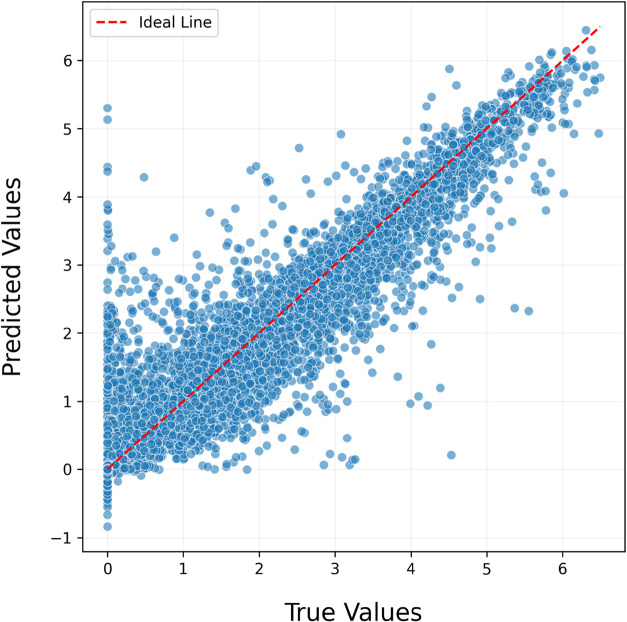
Predicted vs true values plot of the stacking model illustrating the model’s predictive performance. Most data points are clustered around the reference line, indicating that the predicted values are highly consistent with the actual values.

The SHAP (SHapley Additive exPlanations) summary plot illustrates the extent to which the model learns from each feature (Figure 11). In the plot, the feature listed at the top has the greatest influence on the model’s predictions. A concentration of red points toward positive SHAP values indicates that the corresponding feature increases the predicted value, whereas a concentration of blue points in that region indicates a decrease. When red and blue points are positioned on opposite sides of the SHAP value axis, this represents a positive or negative correlation between the feature and the target variable. As seen in the figure, the model primarily learns from the predictions obtained by the CGCNN model. It was found that the band gap value predicted by CGCNN exhibits a positive correlation with the model’s own predictions, while the information indicating whether a crystal is metallic shows a negative correlation. The features in the plot that begin with PCA correspond to the embedding outputs extracted from the CGCNN model, which were reduced using the PCA algorithm and integrated into the training data set.

**Figure 11:**
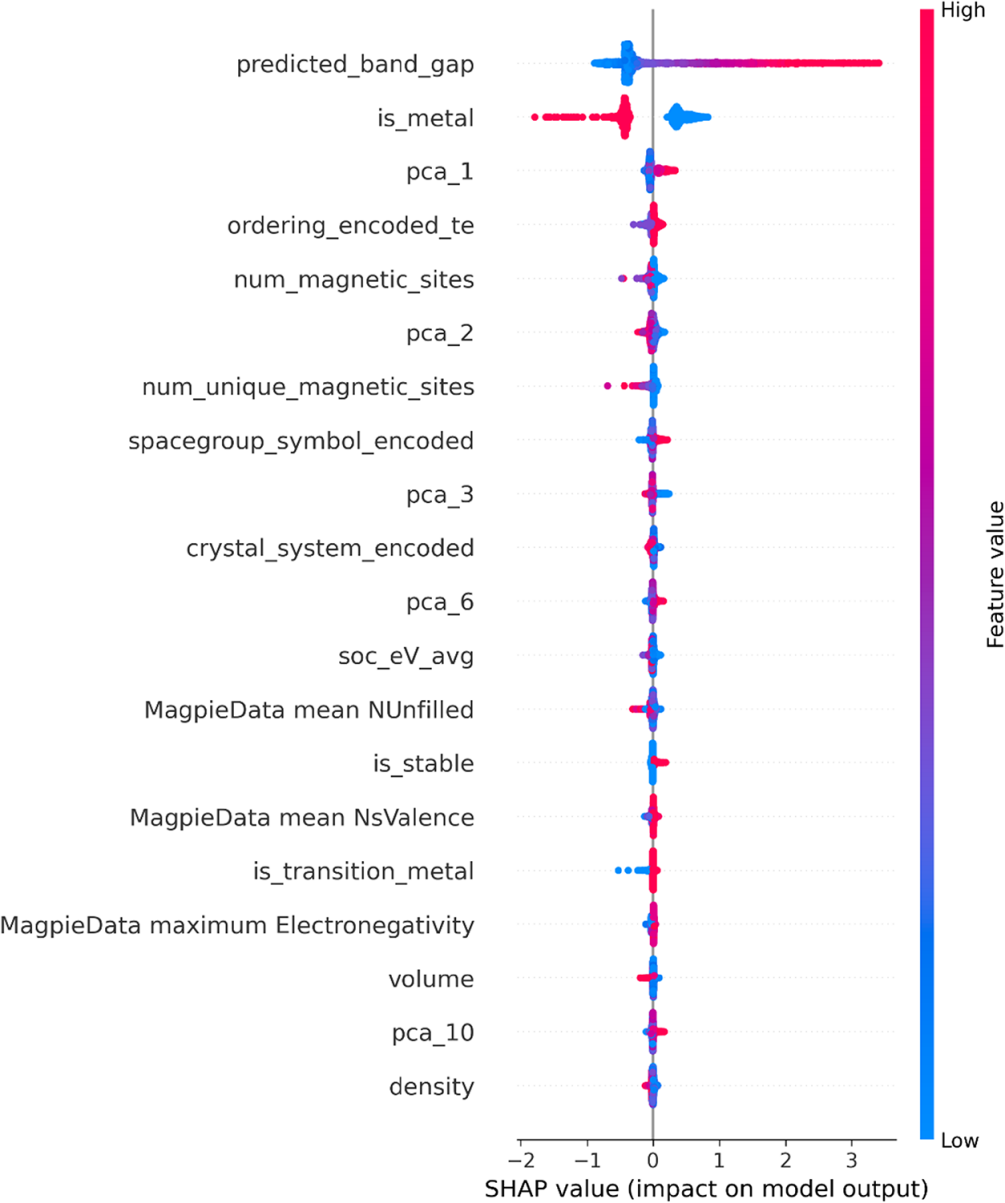
XGBoost SHAP (SHapley Additive exPlanations) analysis of the most influential features: predicted_band_gap, is_metal, and pca_1. This visualization highlights the key features that most strongly influence the XGBoost model’s predictions, with predicted_band_gap, is_metal, and pca_1 having the highest impact on the model’s decision-making process.

The six models trained using the embeddings and prediction scores extracted from the SchNet model, along with their corresponding results, are presented in Table 4. As observed, the best-performing individual model was XGBoost, achieving a *R* ^2^ score of 86%. Various stacking models were then constructed by combining all trained models, and the most successful ensemble was obtained by integrating the FTT, MLP, and CatBoost models under a linear regression framework, achieving a *R* ^2^ of 87.1%, MAE of 0.268, and MSE of 0.244.

**Table 4:** Models Trained on the Data Set Generated Using the SchNet Model and Their Corresponding Results

SCHNET	*R* ^2^	MAE	MSE
XGBOOST	0.869	0.273	0.247
LIGHTGBM	0.863	0.273	0.259
RANDOM FOREST	0.840	0.319	0.303
FTT	0.850	0.270	0.287
MLP	0.851	0.290	0.280
CATBOOST	0.862	0.280	0.618
STACKING MODEL	0.871	0.268	0.244

In the graph, the horizontal axis represents the predictions made by the ensemble model, while the vertical axis shows the difference between the predicted and actual values (error) (Figure 12). The red dashed line indicates zero error. The analysis revealed that 75% of the predictions had errors below 0.517 eV, and 90% had errors below 0.971 eV, providing valuable insights into the model’s overall error distribution. In the 0–4 eV range, the error distribution appears wider; however, the fact that 75% of the errors remain below 0.505 eV indicates that the model produces more consistent and lower-error predictions in low-energy ranges. Conversely, in the 4–6 eV range, although the predictions cluster more closely around the red line (zero-error axis), 75% of the errors fall below 0.704 eV, suggesting that the model exhibits slightly higher error values in this range but still maintains the correct overall trend.

**Figure 12:**
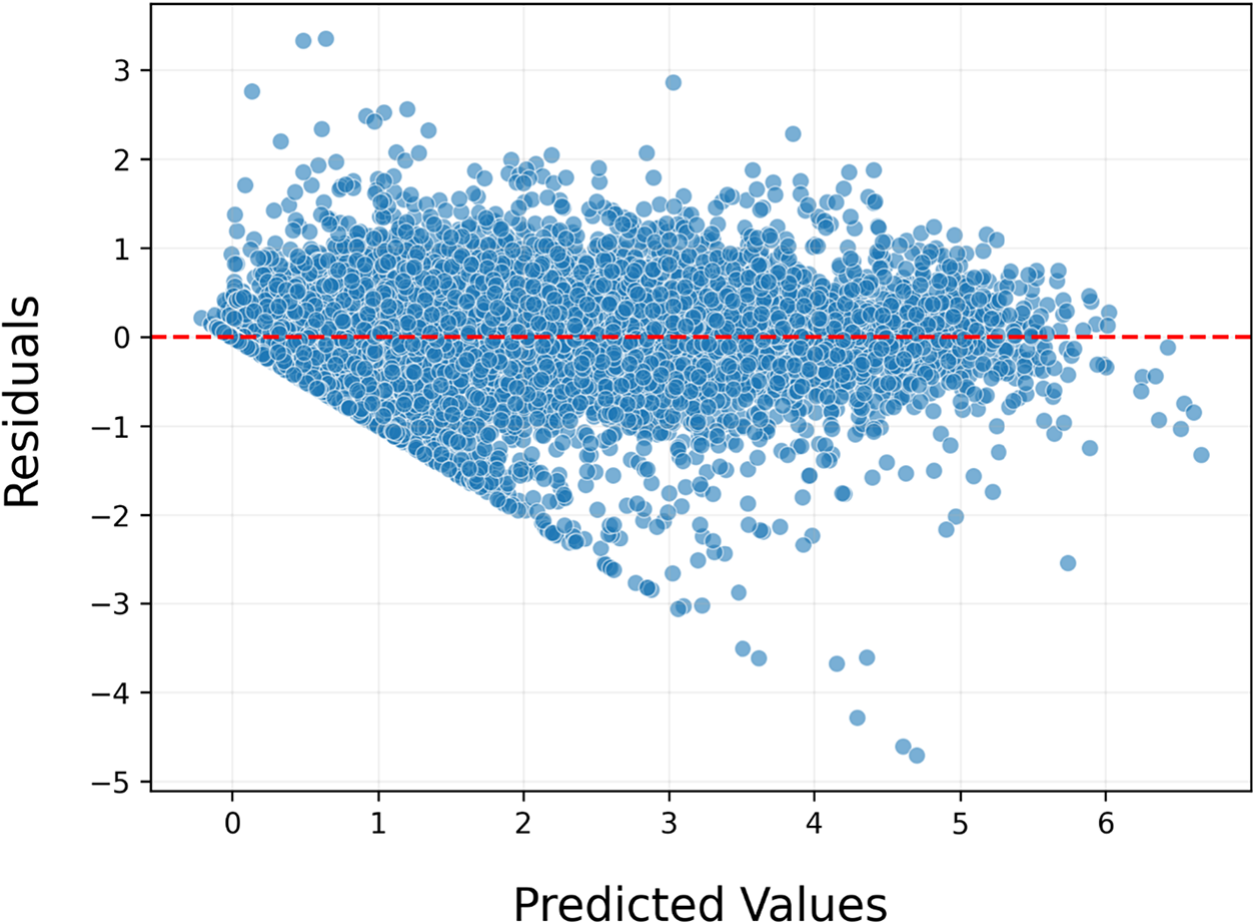
Residuals vs predicted plot of the stacking model, illustrating the distribution of predicted values against error values. The concentration of data points around the red reference line indicates that the model produces balanced and consistent predictions.

In the graph, the horizontal axis represents the predicted values, while the vertical axis shows the actual values (Figure 13). The red dashed diagonal line corresponds to the *Predicted = Actual* line, representing the ideal scenario in which the model’s predictions perfectly match the true values. In a well-performing model, the data points are expected to cluster on or near this diagonal. In our model, the data points are largely concentrated along this line, indicating a strong predictive accuracy. However, it was observed that around the region where the true band gap equals zero, the model produced errors of up to 5.2 eV. The mean of positive errors was calculated as 0.26 eV, while the mean of negative errors was 0.53 eV. The weighted average error across all predictions was −0.001 eV, suggesting a slight tendency of the model to overestimate the true values.

**Figure 13:**
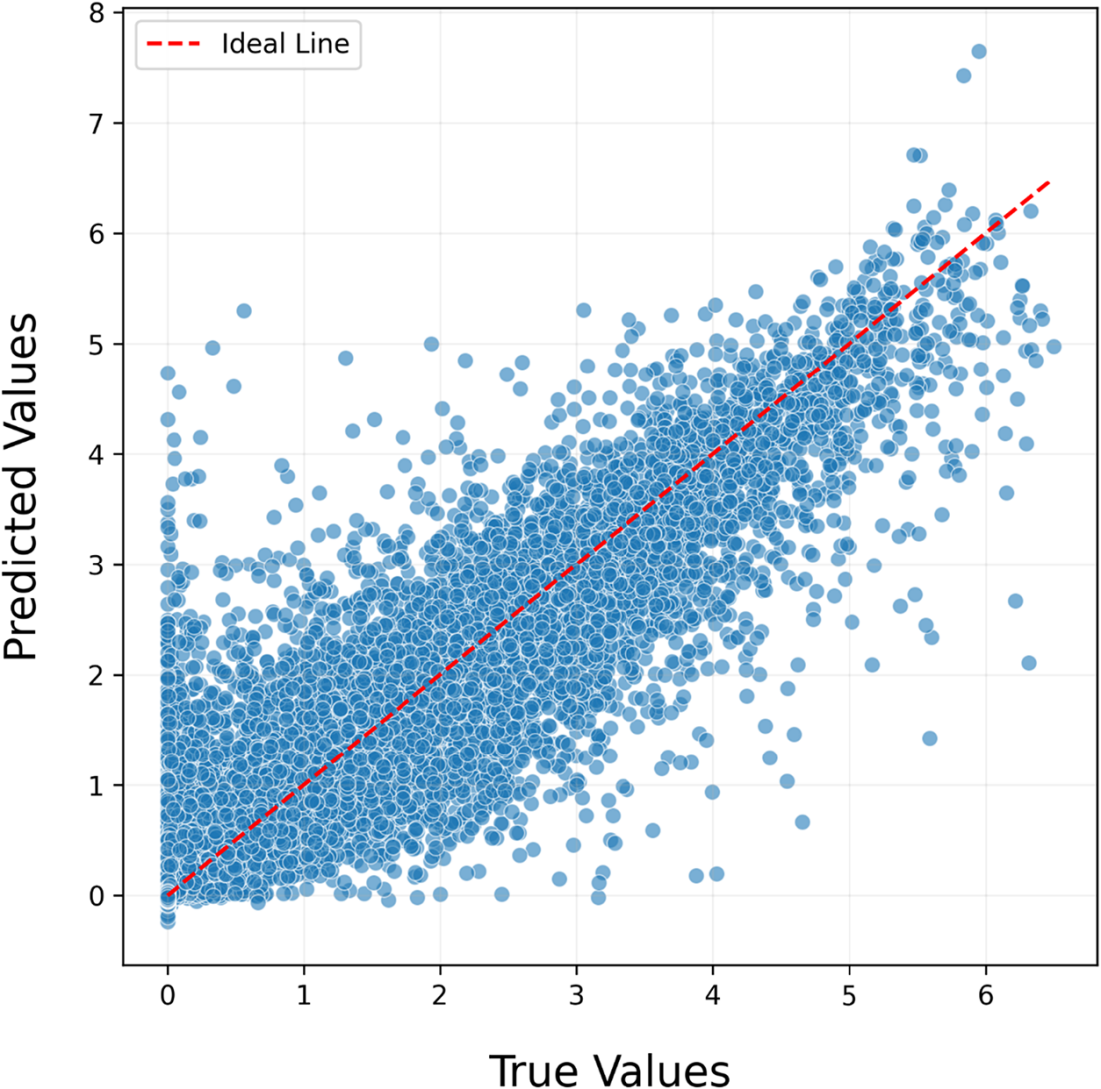
Predicted vs actual values plot of the stacking model. The plot illustrates the relationship between the model’s predicted and true values. The concentration of data points around the diagonal line (error = 0) indicates that the stacking model generally produces accurate and consistent predictions.

Understanding which variables the model learns from is a crucial step in interpreting how the model functions and determining which features have the greatest influence on the band gap. However, since the ensemble model learns from the outputs of other models, it cannot directly reveal which individual features contribute to its learning. Therefore, the analysis was performed using the XGBoost model, which was the best-performing individual model outside of the ensemble. The corresponding SHAP (SHapley Additive exPlanations) summary plot is presented in Figure 15. In the SHAP plot, the feature listed at the top has the greatest influence on the model’s predictions. A concentration of red points toward positive SHAP values indicates an increase in the predicted value, while a concentration of blue points in the same region indicates a decrease. When red and blue points are positioned on opposite sides of the SHAP value axis, this signifies a positive or negative correlation, respectively. As shown in Figure 14, the most critical features for the model are the predictions made by the SchNet model and the information indicating whether the crystal exhibits metallic properties. The analysis revealed a positive correlation between the band gap predictions of the SchNet model and those of the current model, whereas the metallic property information shows a negative correlation. The features beginning with *pca* correspond to the embedding outputs obtained from the SchNet model, which were reduced via the PCA algorithm and integrated into the training data set.

**Figure 14:**
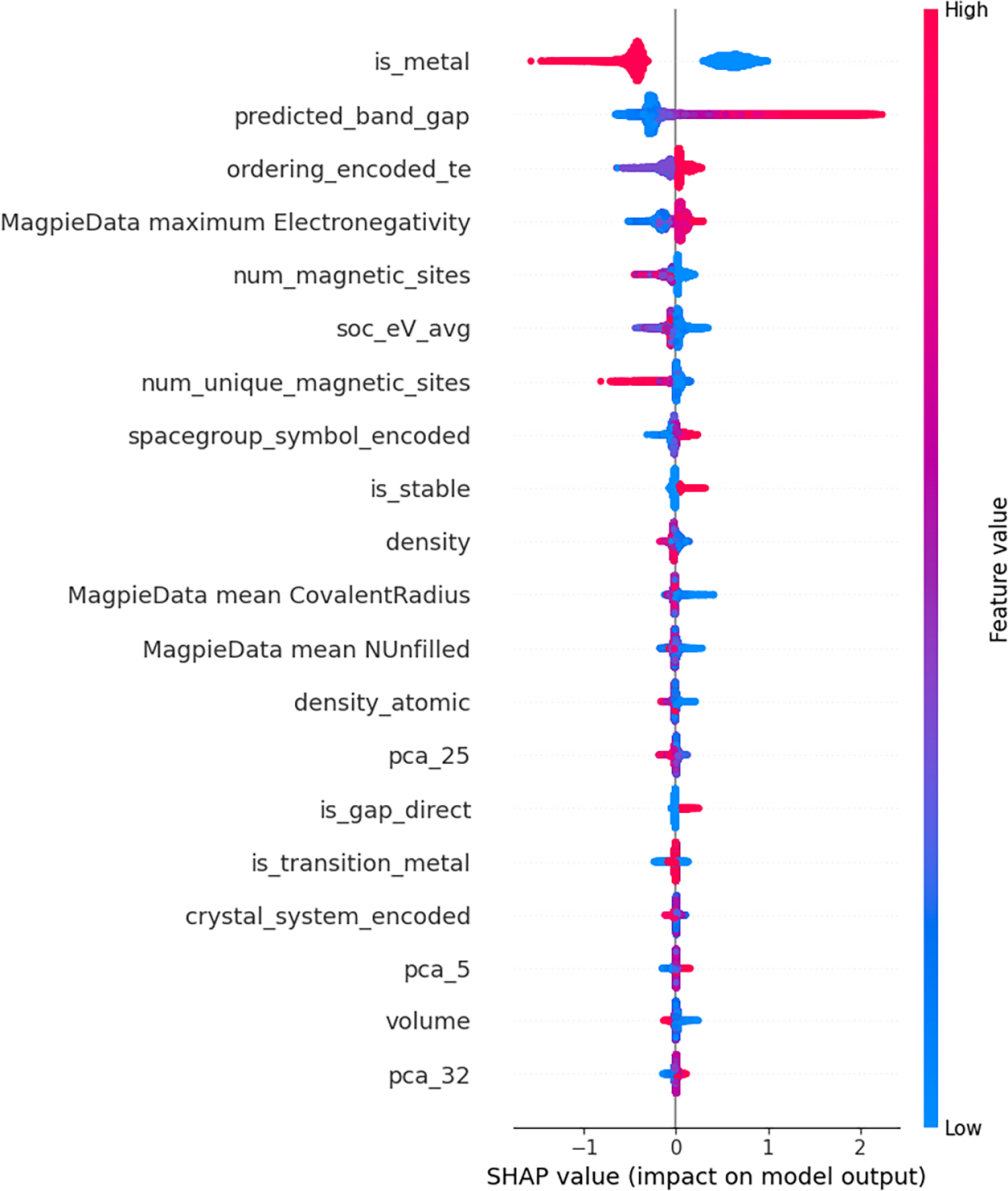
XGBoost SHAP (SHapley Additive exPlanations) analysis of the most influential features: is_metal, predicted_band_gap, and ordering_encoded_te. This visualization highlights the features that contribute most significantly to the XGBoost model’s predictions, with is_metal, predicted_band_gap, and ordering_encoded_te playing a decisive role in the model’s decision-making process.

The performance comparison of the best models trained using the MEGNet, CGCNN, and SchNet frameworks is illustrated in Figure 15. The table presents the *R* ^2^, 1 – MAE, and 1 – MSE scores for each stacking model. Among them, the MEGNet stacking model achieved the highest performance (*R* ^2^ = 0.921, 1 – MAE = 0.8, 1 – MSE = 0.84), followed by the CGCNN stacking model (*R* ^2^ = 0.909, 1 – MAE = 0.787, 1 – MSE = 0.825) and the SchNet stacking model (*R* ^2^ = 0.871, 1 – MAE = 0.732, 1 – MSE = 0.756). These results indicate that the MEGNet framework provides a more accurate and robust prediction performance compared to the other two models

**Figure 15:**
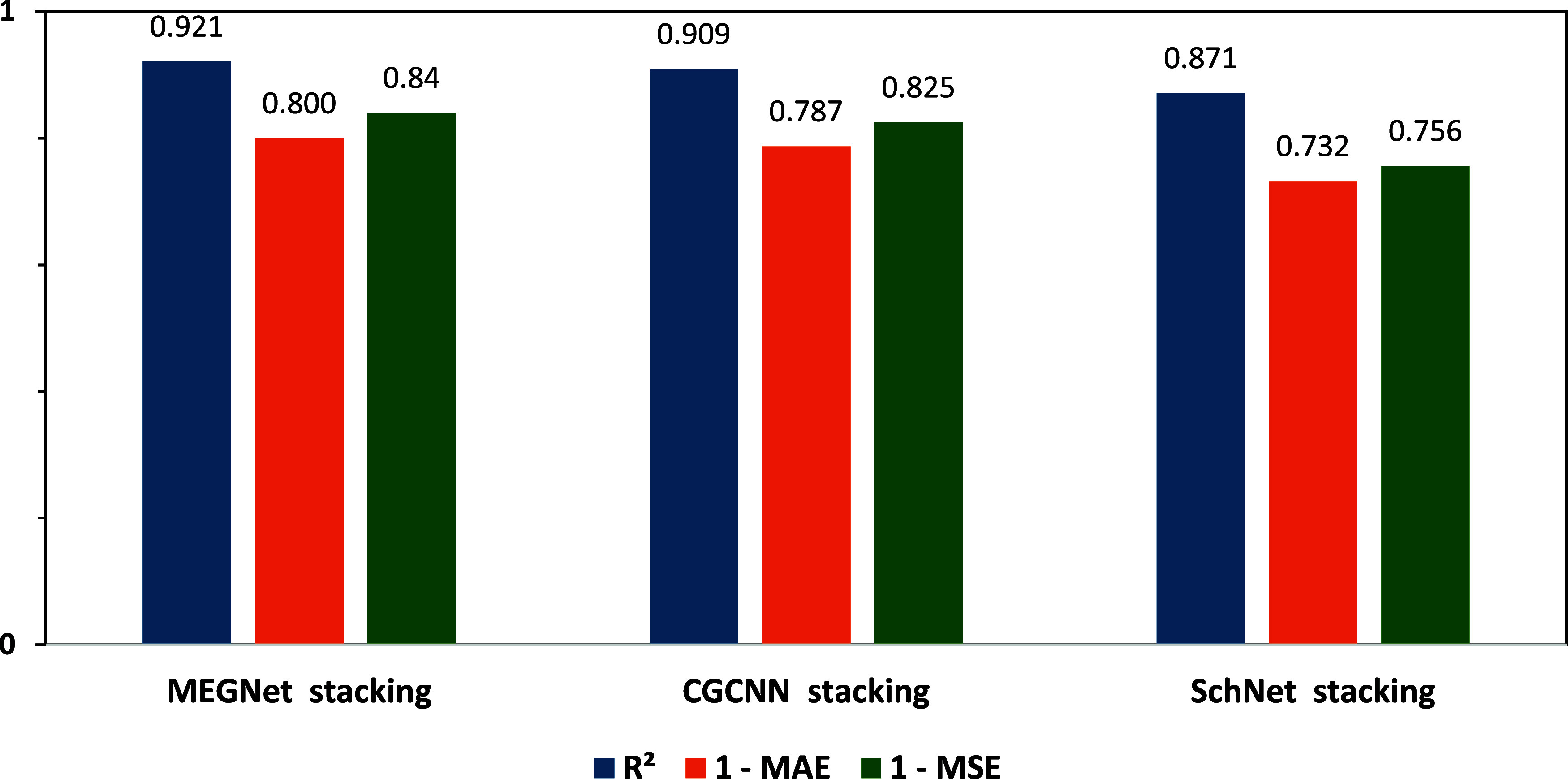
Performance comparison of the best models trained using the MEGNet, CGCNN, and SchNet frameworks.

## 4 Discussion

The findings of this study provide a comprehensive comparison of different machine learning and graph-based deep learning models for predicting material properties. The Crystal Graph Convolutional Neural Network (CGCNN), developed by Xie and Grossman (2018), is one of the first graph-based models designed for this purpose and achieved an *R* ^2^ value of 0.89 using the Materials Project database. By representing atoms as nodes and bonds as edges, CGCNN successfully learned the topological relationships within crystal structures.^37–39^ A more advanced architecture, the Atomistic Line Graph Neural Network (ALIGNN) proposed by Choudhary et al., extended this approach by incorporating second-order bond connections, achieving a higher accuracy of *R* ^2^ = 0.92.^40^ These two studies demonstrated that graph-based deep learning approaches can outperform classical machine learning methods in predicting material properties by directly learning atomic-level interactions.

Among classical approaches, the models developed by Ward et al. using the ICSD and Materials Project databases with Magpie descriptors are notable.^41^ The Multilayer Perceptron (*R* ^2^ = 0.80) and Random Forest (*R* ^2^ = 0.82) achieved moderate accuracy at low computational cost, but their inability to explicitly model crystal topology limited their predictive power compared to graph-based models.^41^

The hybrid model proposed in this study integrates graph-based embeddings (from CGCNN, MEGNet, and SchNet) with physically meaningful crystal metafeatures, forming a multilayered architecture that unites structural and statistical representations. Trained on a large data set containing 136,000 crystal structures, the model achieved *R* ^2^ = 0.921, MAE = 0.191, and MSE = 0.155. This accuracy is statistically comparable to that of the ALIGNN model (Choudhary et al.,) while exhibiting superior generalization capability due to the broader and more diverse data set.^42^ Unlike ALIGNN, which was trained solely on the Materials Project database, the present model encompasses a significantly wider range of crystal chemistries, providing higher scalability and robustness.^42^

Compared to CGCNN, the hybrid model achieved approximately a 3.5% improvement in accuracy and exceeded SchNet-based approaches by more than 6%.^37^ Relative to classical methods, the improvement reached nearly 15%.^40^ This performance enhancement stems from combining the representational power of graph-based embeddings, which capture atomic and topological relationships, with the generalization ability of classical machine learning algorithms. In particular, embeddings derived from the MEGNet model contributed to higher model stability and accuracy by effectively representing global state attributes within crystal structures.^39,40^

Interpretability analysis using SHAP revealed that the most influential features during learning were the embedding-based band gap predictions, the material’s metallic nature, and the number of magnetic atoms.^43^ This finding indicates that the model not only captures numerical correlations but also successfully learns physically meaningful relationships underlying the electronic behavior. As a result, the proposed hybrid framework goes beyond existing studies in terms of both statistical accuracy and physical interpretability.^39–43^

When compared to CGCNN, ALIGNN, and Magpie-based classical approaches, the hybrid model demonstrates a distinctive balance between accuracy, computational efficiency, data scalability, and interpretability.^39–42^ By combining the representational depth of graph-based architectures with the generalization strength of classical models, this approach establishes a scalable and reliable AI-driven framework for materials property prediction and offers a promising foundation for future multiproperty and multisource material discovery systems.

## 5 Conclusions

This study demonstrated that integrating graph-based embeddings with physically meaningful crystal descriptors provides a robust and scalable framework for accurate band gap prediction. The proposed hybrid model, trained on 136,000 crystal structures, achieved a determination coefficient of *R* ^2^ = 0.921, a MAE of 0.191, and a MSE of 0.155, outperforming both classical machine learning and individual graph neural network (GNN) approaches. These results confirm that the fusion of structural representations from CGCNN, MEGNet, and SchNet models with traditional learning algorithms such as XGBoost, LightGBM, and CatBoost enables superior predictive accuracy without relying on direct Density Functional Theory (DFT) outputs. The approach preserves physical interpretability while maintaining computational efficiency, marking a substantial advancement over existing models.

By achieving performance comparable to the state-of-the-art ALIGNN model (*R* ^2^ = 0.92) using a broader and more diverse data set, this framework establishes that combining deep structural embeddings with classical algorithms can achieve both high accuracy and generalization across chemical and structural domains. The SHAP-based interpretability analysis further confirmed that the model effectively learned physically meaningful relationships, where metallicity, magnetic atom content, and embedding-derived band gap predictions emerged as the most decisive features.

The outcomes of this research provide a strong methodological foundation for extending band gap prediction beyond the limits of current data sets and models. Several promising research directions can be pursued to further enhance and generalize the proposed framework. Building upon this framework, future studies can explore multitask learning paradigms that jointly predict multiple physical and electronic properties (e.g., dielectric constant, formation energy, and carrier mobility) or adopt transfer learning techniques using domain-adaptive embeddings across databases such as OQMD, AFLOW, and NOMAD. Additionally, transformer-based architectures and equivariant graph neural networks could be employed to further capture rotational and translational symmetries within crystal structures.

Another promising research direction involves coupling this hybrid predictive framework with inverse materials design and generative models (e.g., diffusion or variational autoencoders) to accelerate the discovery of novel semiconductors and functional materials. Combining explainable AI mechanisms with high-throughput screening pipelines would not only improve predictive confidence but also contribute to a deeper understanding of structure–property correlations at the atomic scale.

In summary, the proposed hybrid model demonstrates that the synergy between graph-based deep representations and classical machine learning offers a powerful, interpretable, and computationally efficient pathway toward next-generation materials informatics. This framework provides a scalable foundation upon which future data-driven materials discovery efforts can be built, adapt, and expand.

## Supplementary Material



## Data Availability

The complete data set used in this study is publicly available at Zenodo: 10.5281/zenodo.18481208. All scripts and computational procedures required to reproduce the results are available at https://github.com/YZE-Crystal/bandgap-prediction

## Notes

Additional information related to the data set preparation, hyperparameter optimization ranges, and supplementary analysis results (PDF)
