# Supplementary material for: Hybrid Graph–Machine Learning Framework for Accurate and Interpretable Band Gap Prediction
Source: J Chem Inf Model. 2026 Mar 19;66(7):3787–802. doi: 10.1021/acs.jcim.6c00365 (PMC13080991; doi:10.1021/acs.jcim.6c00365)
Supplement: Supplementary file 1 [file ci6c00365_si_001.pdf]

Supporting Information

HYBRID GRAPH–MACHINE LEARNING FRAMEWORK FOR ACCURATE AND INTERPRETABLE BAND GAP PREDICTION

Ayhan Aydın[1]*, Ümit Kaya Eryılmaz[2], Onur Bahattin Alkan[3], Pınar Kocagöz[4], Fatih Ekinci[5], Mehmet Serdar Güzel[6]

[1] Department of Computer Engineering, Faculty of Engineering, Ankara University, Ankara, Türkiye
[2] Department of Mathematics, Faculty of Science, Ankara University, Ankara, Türkiye
[3] Department of Computer Engineering, Faculty of Engineering, Ankara University, Ankara, Türkiye
[4] Department of Artificial Intelligence and Data Engineering, Faculty of Engineering, Ankara University, Ankara, Türkiye
[5] Institute of Artificial Intelligence, Ankara University, Ankara, Türkiye
[6] Department of Computer Engineering, Faculty of Engineering, Ankara University, Ankara, Türkiye

Number of pages: 29
Number of figures: 14
Number of tables: 4

Reproducibility and Data Access for:

Hybrid Graph–Machine Learning Framework for Accurate and Interpretable Band Gap Prediction

S1. Public Resources

All data and software required to reproduce the results of this study are publicly available through two permanent repositories:

- **Dataset (archived, DOI versioned):**

  https://doi.org/10.5281/zenodo.18481208

- **Source code and computational pipeline:**

  https://github.com/YZE-Crystal/bandgap-prediction

This separation ensures long-term data preservation (Zenodo) and transparent computational reproducibility (GitHub).

S2. Dataset Description

All crystal structures and corresponding band gap values used in this study were originally obtained from the Materials Project database via its public API.

The exact dataset used for model training, validation, and testing is permanently archived at Zenodo in machine-readable format: **https://doi.org/10.5281/zenodo.18481208**

This dataset contains the precise Materials Project entries and properties used in the manuscript. This guarantees that the dataset used in this study can be accessed without ambiguity and independently of future updates to the Materials Project database.

The dataset archived at Zenodo corresponds exactly to the Materials Project entries selected according to the filtering and preprocessing criteria described in the manuscript, ensuring that the precise training, validation, and test data are permanently preserved without requiring reconstruction from the Materials Project database.

S3. Automatic Data Handling

The GitHub repository contains scripts that demonstrate how the dataset can be programmatically retrieved from the Materials Project using its API. These scripts document the data acquisition procedure used during the study.

After providing a valid Materials Project API key, the scripts automatically download and process the data into the required format.

S4. Data Preprocessing

All dataset cleaning, filtering, and preparation steps are fully defined in:

- preprocessing.ipynb

This notebook produces the processed dataset used for feature extraction and model training.

S5. Structural Feature Extraction (Graph Embeddings)

High-dimensional structural embeddings are extracted from CIF files using three graph neural network models:

- CGCNN
- MEGNet
- SchNet

The extraction procedures are implemented in:

- cgnn_emb_extractor.py
- megnet_emb_extractor.ipynb
- schnet_emb_extractor.ipynb

These scripts reproduce the exact embedding features used in the manuscript.

S6. Machine Learning Models

Individual regression models are trained using:

- CatBoost
- XGBoost
- LightGBM
- Random Forest
- MLP
- FT-Transformer

Training notebooks:

- CatBoost.ipynb
- XGBoost.ipynb
- LightGBM.ipynb
- Random_Forest.ipynb
- MLP.ipynb
- Ftt.ipynb

S7. Stacking Ensemble

The final hybrid ensemble model described in the manuscript is implemented in:

- stack.ipynb

Running this notebook produces the final ensemble predictions reported in the paper.

S8. Environment and Dependencies

All required Python dependencies are listed in:

- requirements.txt

The computational environment used in this study is Python 3.8.

S9. Reproducing the Results

To reproduce the complete results reported in the manuscript, the following execution order is recommended:

1. Obtain the dataset from Zenodo
2. Run preprocessing.ipynb
3. Run the embedding extraction scripts
4. Run the individual model training notebooks
5. Run stack.ipynb to generate the final predictions

This procedure reproduces all tables and figures reported in the manuscript. No additional data collection, manual intervention, or undocumented processing steps are required.
